# Dietary grape pomace extract supplementation improved meat quality, antioxidant capacity, and immune performance in finishing pigs

**DOI:** 10.3389/fmicb.2023.1116022

**Published:** 2023-03-02

**Authors:** Xuekai Tian, Dong Li, Xin Zhao, Zitong Xiao, Jingchun Sun, Tiantian Yuan, Yongcheng Wang, Xinhui Zuo, Gongshe Yang, Taiyong Yu

**Affiliations:** ^1^Key Laboratory of Animal Genetics, Breeding and Reproduction of Shaanxi Province, Laboratory of Animal Fat Deposition and Muscle Development, College of Animal Science and Technology, Northwest A&F University, Yangling, Shaanxi, China; ^2^Qinghai Yufu Animal Husbandry Development Co., Ltd, Qinghai, China; ^3^Ningxia Lilan Winery Co., Ltd, Yinchuan, Ningxia, China

**Keywords:** finishing pigs, grape pomace extract, gut barrier function, inflammation, meat quality

## Abstract

In pig production, reducing production costs and improving immunity are important. Grape pomace, a good agricultural by-product, has been thrown away as food waste for a long time. Recently, we found that it could be used as a new source of pig feed. We investigated the effect of grape pomace on inflammation, gut barrier function, meat quality, and growth performance in finishing pigs. Our results indicated that treatment samples showed a significant decrease in water loss, IL-1β, DAO, ROS, and MDA content (*p* < 0.05). IgA, IgG, IgM, CAT, T-AOC, SOD, and IFN-γ significantly increased compared with those in control samples (*p* < 0.05). Meanwhile, the relative mRNA expression of the tight junction protein occludin showed a significant difference (*p* < 0.05). Analysis of metagenomic sequencing indicated that grape pomace significantly decreased the relative abundance of *Treponema* and *Streptococcus* (*p* < 0.05). In summary, our results demonstrated that grape pomace could improve meat quality, alleviate inflammation, and decrease oxidative stress.

## Introduction

Pigs are one of the most important livestock animals in the world; therefore, it is important to improve the production efficiency in pig production (Cheng et al., 2021; Matarneh et al., 2021). The main part of finishing pig production is giving pigs suitable feed, but the feed cost accounts for 60%–70% of the cost of a large-scale pig farm (Altmann et al., 2019). Many studies reported that reused agricultural by-products could reduce not only the cost but also environmental problems (Reckmann et al., 2016; Altmann et al., 2018, 2019). Previously, antibiotics were usually used to treat and prevent diseases of livestock (Laxminarayan et al., 2013), but their excessive use has caused antibiotic resistance all over the world (Prestinaci et al., 2015; Maier et al., 2021). In recent years, many countries and regions advocated that the use of antibiotics should be reduced in animal production (Angelucci et al., 2019). Meanwhile, the question of how to preserve the health, production, and welfare of pigs without antibiotics has become a serious problem in recent years (Laxminarayan et al., 2013).

Studies reported that the change in microbiota could affect pig’s growth performance, immune performance, and meat quality (Wang et al., 2019; Chen et al., 2021). Therefore, it played a huge role in pig production by improving intestinal health and reducing the use of antibiotics (Bergamaschi et al., 2020; Reverter et al., 2021).

The global annual grape production was about 7.78 billion tons, 57% of which was used for winemaking (Muñoz-Bernal et al., 2021). Grape pomace, a potential feed resource, is mainly composed of peel, seeds, and stems. Statistics from a previous study showed that approximately 13.1 million tons of grape pomace were produced globally every year (Gómez-Brandón et al., 2019). China is one of the largest grape pomace-producing countries, especially northwest China (Supplementary Figure S1). The content of phenolic compounds in grapes pomace is very high, which has cardioprotective properties and enhanced antioxidant capacity (Muñoz-Bernal et al., 2021). However, approximately 70% of the phenolic compounds are left in grape pomace, which is a deplorable amount of waste, and are not being used properly (Muñoz-Bernal et al., 2021).

In our study, we added grape pomace to the finishing period feed of pigs instead of wheat bran. We analyzed the meat quality, immune performance, and intestines of slaughtered pigs, and the meat quality and immune performance of the treatment samples were significantly improved (*p* < 0.05). The sequencing results showed that bacteria *Treponema* and *Streptococcus* played an important role in immune performance. In summary, the use of grape pomace, instead of bran, in the feed not only reduced the production cost but also provided insights into improving the immune performance of pigs in the absence of antibiotics.

## Materials and methods

All animal studies were approved by the Institutional Animal Care and Use Committee of Northwest A&F University (approval number: NWAFU-314021167). All operations were carried out in accordance with the university guidelines for animal research. All pigs were cared for and sacrificed following the guidelines of the Institutional Animal Care and Use Committee of Northwest A&F University (Yangling, China).

### Experimental animals and diet

A total of 24 fellow male pigs (Guanzhong Black Pig × Landrace) selected from 223 male pigs, with similar body weights (initial body weight 55.14 ± 0.59 kg), were randomly divided into two groups (six pens per group and two pigs per pen) to undergo a 75-day feeding period. All diets were based on the National Research Council (de Lange, 2013), and all diets were processed by Anyou Feed Company (Yangling, China; Supplementary Tables S1, S2). Animals in the control group received a basal diet with no additives. Each animal in the treatment group received a basal diet that replaced 6% wheat bran with 6% dried grape pomace powder. The pigs had *ad libitum* access to feed and water.

### Sample collection

From the beginning of feeding the grape pomace, fecal samples of each pig were collected every 25 days. At the end of the experiment, six pigs were randomly chosen from each group and then slaughtered and exsanguinated. At the end of the experiment, the final body weight, daily feed intake, daily weight gain, and feed conversion rate were measured. Before slaughter, the pigs (one pig in one pen) had free access to water. The pigs were slaughtered by electrical stunning, exsanguinated, dehaired, peeled, eviscerated, and split down the midline in compliance with the Chinese guidelines (Standardization Administration of the People’s Republic of China, 2006). The carcass weight was measured to calculate the dressing percentage. Samples of the longissimus thoracis (LT) were collected for the analysis of meat quality and the metabonomics of amino acids and medium- and long-chain fatty acids.

### Meat quality

The meat quality was measured as described previously (Altmann et al., 2019; Cheng et al., 2021; Matarneh et al., 2021). The pH and meat color CIE LAB value (L*, a*, and b*) of the LT were determined at the 10th rib at 45 min post-mortem, using a portable pH meter (HI99163N, Hana, Italy) and a colorimeter (Konica Minolta Inc., Tokyo, Japan). Before measurement, the pH meter was calibrated with a standard pH buffer (pH 4.0, 7.0, and 10.0), and the colorimeter was also calibrated (Matarneh et al., 2021). Two repeated measurements were performed for pH and meat color, and the mean was used for further data analysis. Cooking loss was calculated based on weight loss and presented as the weight change percentage. Approximately 15 g of the sample was weighed and immersed in a water bath at 80°C until the internal temperature reached 75°C. Afterward, the bags were cooled to 25°C, wiped, and reweighed. The shear force of the left longissimus thoracis (diameter: 1 cm and thickness: 1 cm) was measured using a tenderometer (C-LM3, Northeastern University, Shenyang, China). There were three positions in the cooked meat that were randomly selected.

### Amino acid and fatty acid contents

The targeted metabolomics profiling was performed as described in the previous study (Altmann et al., 2018; Maier et al., 2021). Five LT samples were used for the analysis of amino acid and fatty acid contents per group. Ultra-high-performance liquid chromatography coupled with the tandem mass spectrometry (UHPLC–MS/MS) system (ExionLC™ AD UHPLC-QTRAP 6500+, AB SCIEX Corp., Boston, MA, United States) was used to quantitate amino acids in Novogene Co., Ltd. (Beijing, China). Separation was performed on an ACQUITY UPLC BEH Amide Column (2.1 × 100 mm, 1.7 μm) which was maintained at 50°C. The mobile phase, consisting of 0.1% formic acid in 5 mM ammonium acetate (solvent A) and 0.1% formic acid in acetonitrile (solvent B), was delivered at a flow rate of 0.30 ml/min. The solvent gradient was set as follows: initial 80% B, 0.5 min; 80%–70% B, 2 min; 70%–45% B, 4 min; 45%–80% B, 6 min; and 80% B, 9 min. The mass spectrometer was operated in positive multiple reaction mode (MRM). Parameters were as follows: ionspray voltage (5,500 V), curtain gas (35 psi), ion source temp (550°C), and ion source gas of 1 and 2 (50 and 60 psi). The differentially expressed amino acid and fatty acid were identified with a *p*-value of <0.05 and a fold-change of >2 between the two groups.

### RNA-Seq analysis

The RNA-Seq was measured as described previously (Angelucci et al., 2019). Total RNA was extracted from control and treatment LT tissues. The transcriptome sequencing experiments were performed by Novogene Company (Beijing, China). The transcriptome library for sequencing was generated using a KAPA-Stranded RNA-Seq Library Prep Kit (Illumina California, U.S.A) following the manufacturer’s recommendations. The clustering of the index-coded samples used the KAPA RNA Adapters set1/set2 for Illumina. After clustering, the libraries were sequenced on the Illumina HiSeq X Ten platform using a (2 × 150 bp) paired-end module (Reckmann et al., 2016). The differentially expressed genes were identified with a *p*-value of <0.05 and a fold-change of >2 between the two groups.

### Measurement of inflammatory cytokines and antioxidant indices in serum

The antioxidant indices and inflammatory cytokines were measured as described in the study (Laxminarayan et al., 2013). Antioxidant capability and immune performance were measured using serum samples. In short, T-AOC, T-SOD, CAT, and MDA used respective assay kits to analyze, and all steps followed the kit instructions provided by the manufacturer (Hengyuan Biotechnology, Shanghai, China). Immunoglobulin G (IgG), immunoglobulin M (IgM), immunoglobulin A (IgA), interferon-γ (IFN-γ), interleukin-1 beta (IL-1β), interleukin-6 (IL-6), interleukin-10 (IL-10), diamine oxidase (DAO), and endothelin (ET) were analyzed using the kit instructions provided by the manufacturer (Hengyuan Biotechnology, Shanghai, China).

### Hematoxylin and eosin staining

The Hematoxylin and eosin (H&E) staining of intestinal segments was performed as described in the previous study (Prestinaci et al., 2015). The fixed intestinal segments were dehydrated, then embedded in paraffin, and cut into 5-μm thick sections. The sections were departmentalized, rehydrated, and stained with H&E. At least five images per section were obtained. The villus height and crypt depth were determined on the images using a microscope (Olympus, Tokyo, Japan).

### RT-qPCR analysis

The mRNA expression of tight junction proteins, including ZO-1, occludin, and claudin-1, was determined in ileum mucosa by real-time quantitative PCR as described in the study of Hu et al. (Prestinaci et al., 2015) Total RNA was extracted from the ileum mucosa or spleen using TRIzol reagent (Sigma-Aldrich, Saint Louis, MO, United States). The concentration and integrity of RNA were determined by a nucleic acid/protein analyzer (Beckman Coulter DU800, Beckman Coulter Inc., Fullerton, CA, United States). An amount of 1 μg RNA was reverse-transcribed into cDNA by using the Prime Script RT Reagent Kit (Takara Biomedical Technology). PCR amplification was performed using the SYBR PCR mix (Takara Biomedical Technology Co). The primers for quantitative PCR are listed in Supplementary Table S1.

### DNA extraction and 16S rRNA sequencing

The extraction of DNA from cecal contents and 16S rRNA gene sequencing were performed as described in the study of Prestinaci et al. Total genome DNA and 16S rRNA from stool samples were extracted using the CTAB/SDS method. DNA concentration and purity were monitored on 1% agarose gel. According to the concentration, DNA was diluted to l ug/μL using sterile water. The amplification and sequencing of 16 s rRNA were commissioned by Biomarker Biotechnology Co., Ltd. (Beijing, China) for testing. Paired-end reads were assigned to samples based on their unique barcode and truncated by cutting off the barcode and primer sequence. Paired-end reads were merged using FLASH (VI.2.7, http://ccb.jhu.edu/software/FLASH/). QIME2 (version 2017.6.0) was used for bioinformatics analysis (Bolyen et al., 2019). There were 12 samples per group used for our study.

### Statistical analysis

Data were analyzed by one-way ANOVA with Statistical Product and Service Solutions (SPASS 21.0 for windows; SPASS Inc., Chicago, IL, United States) software, followed by Duncan’s multiple range test. The results were expressed as the mean ± SD. Correlations were analyzed by using spearman correlation analysis. A *p*-value of <0.05 was considered significantly different.

## Results

### Grape pomace improved meat quality in finishing pigs

**Figure 1:**
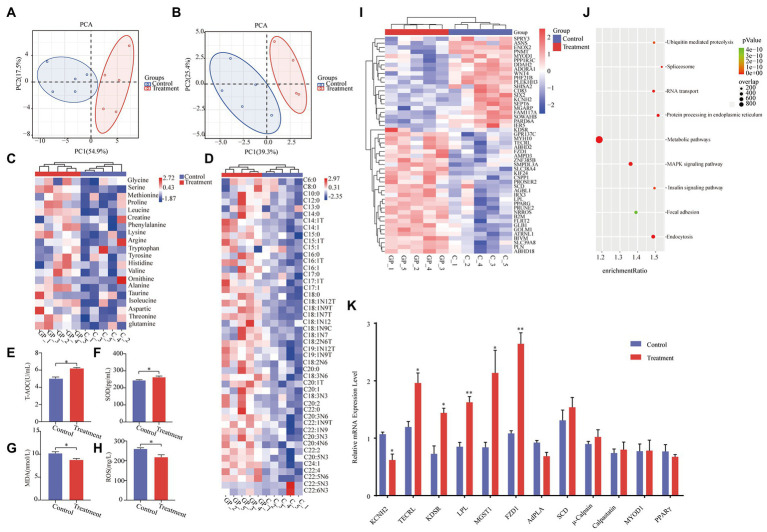
Effect of grape pomace diet on the meat quality. **(A)** Principal component analysis ordination plots of amino acid in the control and treatment group based on the Bray–Curtis distance metric. **(B)** Principal component analysis ordination plots of fatty acid in the control and treatment groups based on the Bray–Curtis distance metric. **(C)** Heatmap of amino acid in the control and treatment groups. **(D)** Heatmap of fatty acid in the control and treatment groups. Meat levels of indicators including **(E)** T-AOC, **(F)** SOD, **(G)** MDA, and **(H)** ROS were determined by using respective kits. Control group, a basal diet; treatment group, a basal diet’s wheat bran is replaced with grape pomace. Data were shown as means ± SD (*n* = 5), * refers to a significant difference (*p* < 0.05). T-AOC, total antioxidant capacity; SOD, superoxide dismutase; MDA, malondialdehyde; and ROS, reactive oxygen species. **(I)** Heatmap of transcripts in the control and treatment groups. **(J)** Gene enrichment analyses of the significantly changed pathways. **(K)** RT-qPCR analysis of KCNH2, TECRL, KDSR, LPL, MGST1, FZD1, AdPLA, Scd, μ-Calpain, Calpastatin, MYOD1, and PPARγ in LT from the control group and treatment group. *p < 0.05, **p < 0.01.

As shown in Table 1, there were no differences in growth performance (initial weight, final weight, ADG, ADFI, and F:G) and carcass (hot carcass weight, dressing percentage, and lion muscle area; *p* > 0.05) between the treatment group and the control group. The treatment group’s water loss (24 and 48 h), drip loss (24 and 48 h), and cooking loss were significantly lower than those in the control group (*p* < 0.05, Table 2). Principal component analysis (PCA) showed differences in amino acid content and fatty acid content (Figures 1A,B). Alanine in the treatment group was significantly higher than that in the control group (*p* < 0.05, Figure 1C). In addition, the contents of C14:1 T, C18:0, C18:2 N6, and C20:4 N6 in the treatment group were significantly higher than those in the control group (*p* < 0.05, Figure 1D).

**Table 1:** Effect of grape residue meal on carcass traits of longissimus thoracis in pigs.

Items	Control	Treatment
Initial weight (kg)	54.72 ± 3.73	55.56 ± 4.30
Final weight (kg)	115.28 ± 6.48	115.11 ± 10.19
ADG (kg)	0.71 ± 0.06	0.72 ± 0.10
ADFI (kg)	2.48 ± 0.17	2.38 ± 0.27
F:G (kg/kg)	3.52 ± 0.39	3.34 ± 0.41
Hot carcass weight (kg)	87.74 ± 5.09	88.60 ± 7.56
Dressing percentage (%)	76.13 ± 2.00	77.05 ± 3.31
Loin muscle area (cm^2^)	30.98 ± 6.21	29.06 ± 4.31

a, b means in the same row with no superscript letters after them or with a common superscript letter following them are not significantly different (*p* > 0.05). Values are expressed as the means ± SD (*n* = 6); ADG, daily weight gain, ADFI, average daily feed intake, F:G, feed conversion ratio.

**Table 2:** Effect of grape residue meal on quality traits of longissimus thoracis in pigs.

Items	Control	Treatment
pH_45min_	6.63 ± 0.13	6.78 ± 0.10
L*_45min_	44.52 ± 1.19	45.06 ± 1.34
a*_45min_	8.15 ± 0.69	8.21 ± 0.48
b*_45min_	15.42 ± 0.33	15.78 ± 1.02
pH_24h_	5.58 ± 0.09	5.68 ± 0.03
L*_24h_	53.51 ± 0.94	54.50 ± 0.91
a*_24h_	11.37 ± 2.27	12.72 ± 1.11
b*_24h_	19.60 ± 0.98	19.61 ± 1.40
Water loss_24h_ (%)	32.90 ± 2.81^a^	24.82 ± 1.33^b^
Drip loss_24h_ (%)	2.54 ± 0.19^a^	1.49 ± 0.10^b^
Water loss_48h_ (%)	38.03 ± 1.73^a^	33.31 ± 2.02^b^
Drip loss_48h_ (%)	7.16 ± 0.49^a^	4.32 ± 0.23^b^
Share force (*N*)	31.81 ± 7.66	39.63 ± 6.37
Cooking loss (%)	35.25 ± 0.83^a^	32.05 ± 0.79^b^

a, b means in the same row with no superscript letters after them or with a common superscript letter following them are not significantly different (*p* > 0.05). Values are expressed as the means ± SD (*n* = 6).

The activity of T-AOC and SOD in the treatment group was significantly higher than that in the control group (*p* < 0.05, Figures 1E,F). Meanwhile, the MDA content and ROS activity in the treatment group were significantly lower than those in the control group (*p* < 0.05, Figures 1G,H).

RNA sequencing revealed that 295 transcripts were significantly changed in LT from finishing pigs (Figure 1I). Among these changed transcripts were the genes involved in energy metabolism, including the metabolic pathways, the MAPK signaling pathway, and the insulin signaling pathway (Figure 1J). The significant changes in KCNH2 TECRL, KDSR, LPL, MGST1, and FZD1 in LT samples of finishing pigs were confirmed by real-time quantitative PCR (RT-qPCR) analyses (Figure 1K).

### Grape pomace attenuated oxidative stress and inflammation in finishing pigs

**Figure 2:**
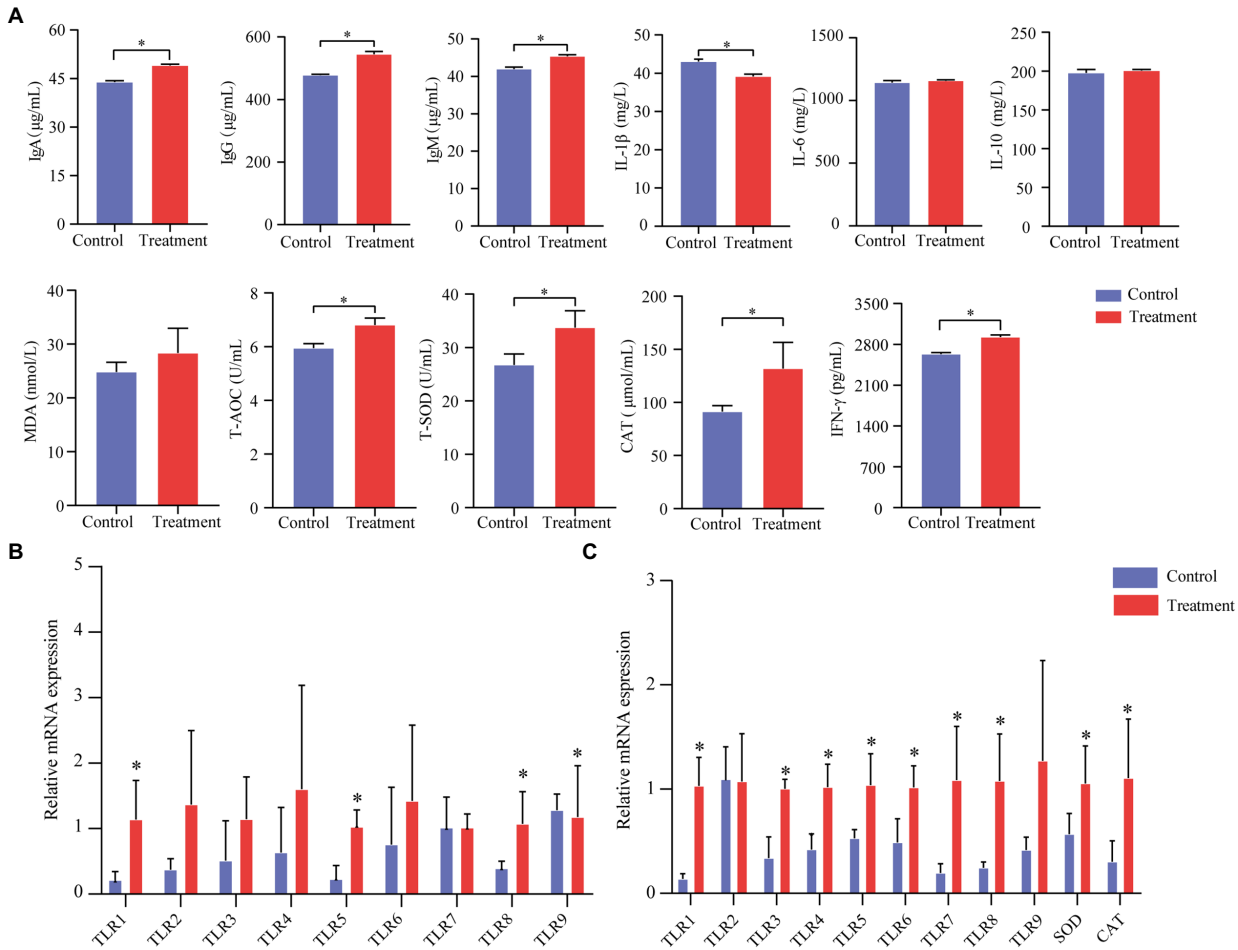
Effect of grape pomace on antioxidant indicators and the production of inflammatory cytokines of finishing pigs. **(A)** Serum levels of indicators including T-AOC, T-SOD, CAT, MDA, IgA, IgG, IgM, IFN-γ, IL-1β, IL-6, and IL-10 were measured by using ELISA kits (*n* = 6). **(B)** mRNA expression of immune-related genes in the ileum mucosa of finishing pigs (*n* = 6). **(C)** mRNA expression of immune-related genes in the spleen of finishing pigs (*n* = 6). Control group, a basal diet; treatment group, a basal diet’s wheat bran is replaced with grape pomace. Data were shown as means ± SD, * refers to significant difference (*p* < 0.05). Ig, immunoglobulin; CAT, catalase; TLRs, Toll-like receptors.

We measured total superoxide dismutase (T-SOD), total antioxidant capacity (T-AOC), malondialdehyde (MDA), and catalase (CAT) to evaluate the oxidative status of finishing pigs (serum). Serum cytokines plays an important role in immunity, which including IL-1β, IL-6, IL-10 and IFN-γ. As was shown in Figure 2, the activities of T-AOC, T-SOD, and CAT in the treatment group were significantly increased (*p* < 0.05, Figure 2A). The MDA content of the treatment group showed no differences (*p* < 0.05, Figure 2A). IgA, IgG, IgM, and IFN-γ significantly improved in the treatment group (*p* < 0.05, Figure 2A). IL-1β in the treatment group was significantly lower (*p* < 0.05, Figure 2A). Besides, there were no differences in IL-6 and IL-10 between the treatment group and the control group in finishing pigs (*p* > 0.05).

To further describe immune performances, we analyzed the immune-related mRNA expression level of the spleen and the ileum. The treatment group’s TLR-1, TLR-3, TLR-4, TLR-5, TLR-6, TLR-7, TLR-8, SOD, and CAT mRNA expression levels were significantly increased compared with the control group (*p* < 0.05, Figure 2B). Meanwhile, we found that TLR-1, TLR-5, and TLR-8 in the treatment group were significantly higher than those in the control group (*p* < 0.05, Figure 2C).

### Grape pomace enhanced the expression of tight junction protein occludin

**Figure 3:**
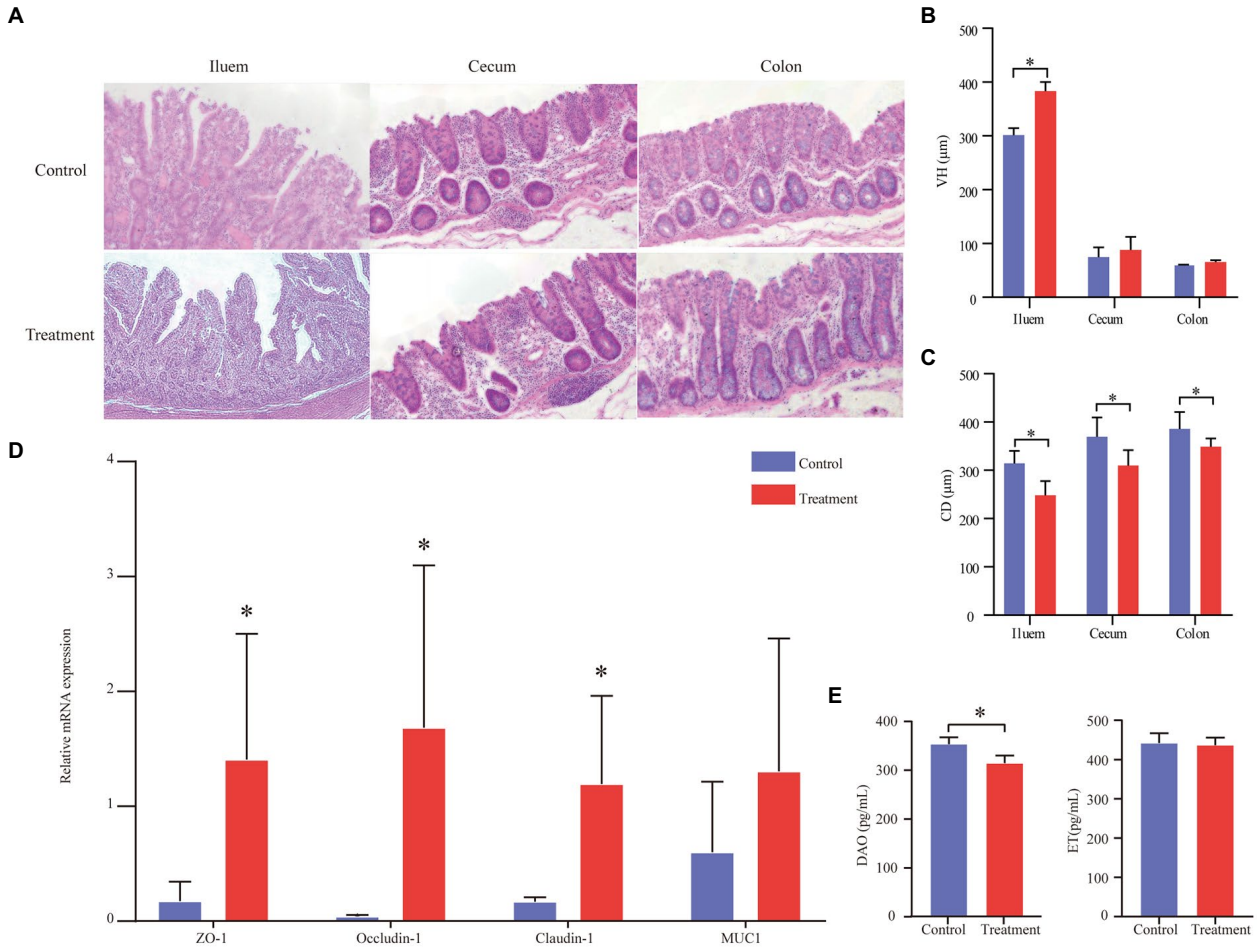
Effect of grape pomace diet on the expression of tight junction proteins of finishing pigs. **(A)** Effects of grape pomace dietary on the intestinal morphology of finishing pigs. **(B)** VH of ileum, cecum, and colon (*n* = 6). **(C)** CD of ileum, cecum, and colon (*n* = 6). **(D)** The mRNA expression of ZO-1, Occludin-1, Claudin-1, and MUC1 was quantitated by real-time PCR (*n* = 6). Control group, a basal diet; treatment group, a basal diet’s wheat bran is replaced with grape pomace. Data were shown as means ± SD, * refers to significant difference (*p* < 0.05). VH, villous height; CD, crypt depth; DAO, diamine oxidase; ET, endothelin; ZO-1, zonula occludens 1; and MUC1, Mucin 1; **(E)** Serum DAO and ET content (*n* = 6).

The data on intestinal morphology are shown in Figure 3A. The ileum and the colon’s villous height (VH) of the treatment group significantly increased (*p* < 0.05, Figure 3B). The crypt depth (CD) of the treatment group significantly decreased compared with the control group (*p* < 0.05; Figure 3C). In addition, the expression of tight junction proteins, including ZO-1, occluding-1, claudin-1, and MUC1, was measured by real-time PCR and immunohistochemical staining to assess the intestinal mechanical barrier function (Figure 3D). We found that the treatment group’s expression of ZO-1, occludin-1, and claudin-1 was significantly higher than the control group’s expression (*p* < 0.05, Figure 3D). In addition, the treatment group’s DAO content was significantly higher than that of the control group (*p* < 0.05, Figure 3E). The ET content between the treatment group and the control group had no differences (*p* > 0.05, Figure 3E).

### Grape pomace improved intestinal health

**Figure 4:**
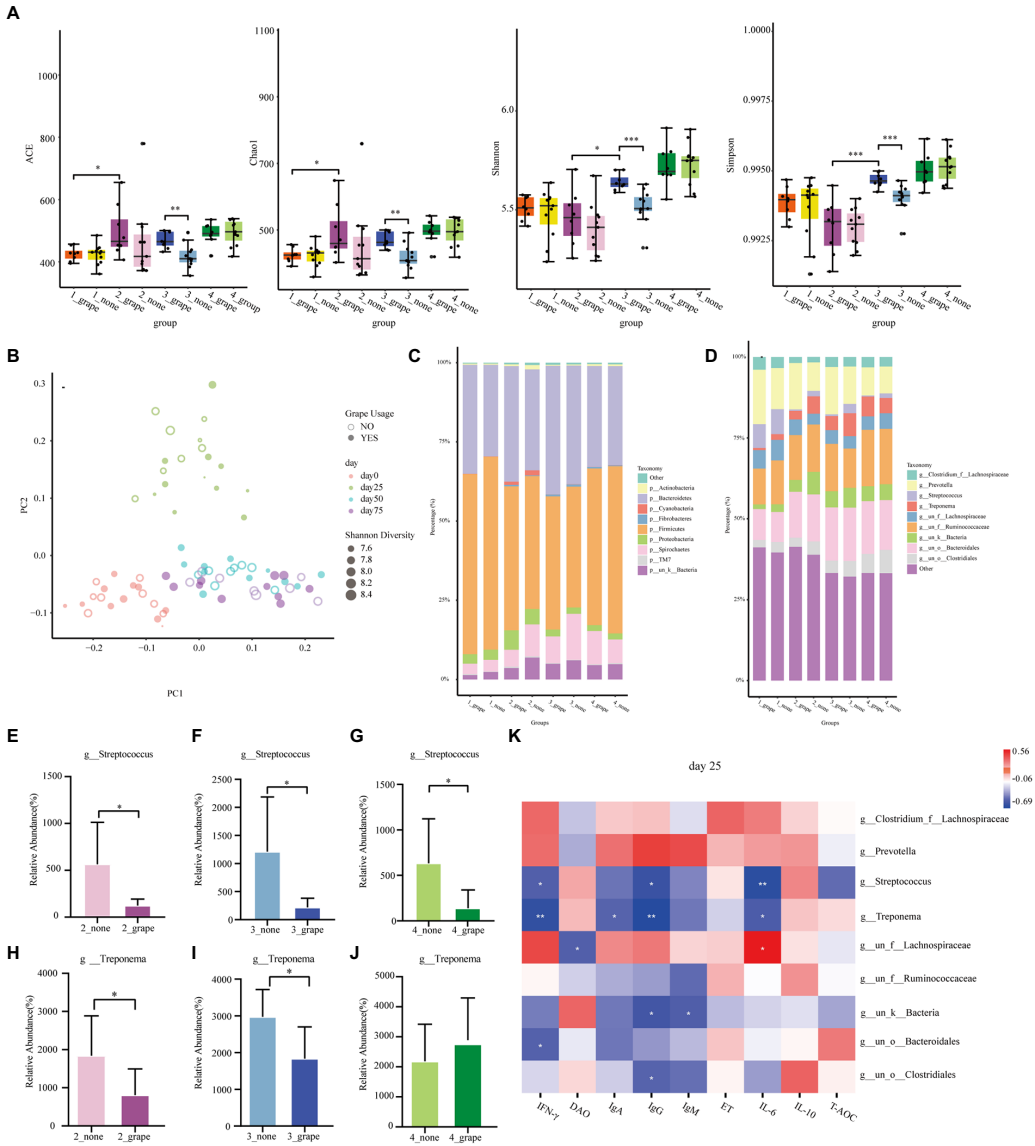
Effect of grape pomace diet on the microbial community of finishing pigs. **(A)** Effect of grape pomace diet on the Chao1, Shannon, ACE, and Simpson index. **(B)** Analysis of similarities (ANOSIM) between control and treatment groups. **(C)** The relative abundance of cecal microbial at the phylum level (mean of each group). **(D)** The relative abundance of cecal microbial at the genus level (mean of each group). Effect of grape pomace diet on the relative abundance of the microbial family *g_Streptococcus* on **(E)** day 25, **(F)** day 50, and **(G)** day 75. Effect of grape pomace diet on the relative abundance of microbial family *g_Treponema* on **(H)** day 25, **(I)** day 50, and **(J)** day 75. **(K)** Heatmap of the significant differences of family correlated with the antioxidant indicators and cytokines levels on day 25.

A total of 72 fecal samples were used in 16S rRNA sequence analysis. The Shannon index, the Chao1 index, the Simpson index, and the ACE index of the treatment group were significantly higher than those of control group on day 50 (*p* < 0.05, Figure 4A). There was no difference between the control group and the treatment group on days 0, 25, and 75 (*p* > 0.05). According to the PCoA result, the gut microbiota of finishing pigs showed a difference between the treatment group and the control group (Figure 4B). There were no differences between the treatment group and the control group at the phylum level (*p* > 0.05, Figure 4C), but there were differences at the genus level (*p* < 0.05, Figure 4D). The relative abundance of *Streptococcus* was significantly decreased in the treatment group on days 25, 50, and 75 (*p* < 0.05, Figures 4E–G). In addition, on days 25 and 50, the relative abundance of *Treponema* significantly decreased in the treatment group (*p* < 0.05, Figures 4H–J). We carried out correlation analysis on some bacterial genera (*Prevotella*, *Streptococcus*, *Treponema*, *Lachnospiraceae*, etc.) and serum indicators. On day 25, *Treponema* had a negative linear correlation with IgG, IFN-γ, IgA, and IL-6 (*p* < 0.05), and *Streptococcus* had a negative linear correlation with IgG, IFN-γ, and IL-6 (*p* < 0.05, Figure 4K). On day 50, *Treponema* had a negative linear correlation with IgG, IFN-γ, IgA, and IL-6 (*p* < 0.05), and *Streptococcus* had a negative linear correlation with IgA and IL-6 (*p* < 0.05, Supplementary Figure S2A). Meanwhile, *Streptococcus* had a negative linear correlation with IgA on day 75 (*p* < 0.05, Supplementary Figure S2B).

## Discussion

Foodstuffs are limited by land, especially in the case of animal protein production. As the population grows, the cultivated land area *per capita* decreases sharply (Bai et al., 2021). To meet the increasing animal protein demands, reducing the cost is very important in livestock production (Kim et al., 2019). Some studies reported that reusing agriculture by-products could reduce production costs and alleviate environmental pressure (Altmann et al., 2018, 2019). Grape pomace is one of the agricultural by-products whose main components were phenolic compounds that could improve antioxidant capacity, prevent platelet aggregation, change blood lipids, promote vasodilation, and reduce inflammation (Muñoz-Bernal et al., 2021); however, the high annual yield of high-quality grape pomace has been underdeveloped (Muñoz-Bernal et al., 2021).

Grape pomace was similar to wheat bran in the nutrition content, but grape pomace had more fiber (Supplementary Table S2), which could influence finishing pigs’ growth performance (Zhou et al., 2016). Surprisingly, we found that the wheat bran in the diet replaced with grape pomace did not have negative effects on finishing pigs’ growth performance. The high ratio of VH/CD in the intestine was regarded as a marker of the improved nutrient digestion-absorption capacity (Biasato et al., 2019), and our result showed that this may be one reason why the treatment group and the control group had no differences. Meat quality is one of the main evaluation indicators of animal protein sources; our result showed that the treatment group’s juiciness was significantly improved, which might have a positive effect on consumer preferences (Jing et al., 2014). Phenolic compounds could improve meat quality by improving the antioxidant enzyme system and delaying lipid peroxidation (Mu et al., 2020; Jin et al., 2021). Our results showed that grape pomace improved meat quality by improving meat antioxidant capacity and decreasing lipid peroxidation. Studies showed that a diet supplemented with alanine could facilitate muscle growth, strength, and power (Kopec et al., 2020). Our results showed an increase in alanine in the treatment group, which was beneficial to human health (Ron-Harel et al., 2019). In addition, the composition of the meat fatty acid played an important role in meat quality (antioxidant, flesh color, flavor, etc; Wood et al., 2008). Many reports found that saturated fatty acids (SFAs) were closely related to modern diseases including cancers and coronary heart disease (Wood et al., 2008). Meanwhile, polyunsaturated fatty acid (PUFA) was reported to decrease cholesterol and triglycerides, which was beneficial to human health (Ruiz-Núñez et al., 2016). C18:2 N6 has been reported as an important polyunsaturated fatty acid for our health, and studies showed that it could produce meat with better quality (de Oliveira et al., 2017; Erasmus and Hoffman, 2018). In our study, the C18:2 N6 content of the treatment group was increased significantly (*p* < 0.05). Meanwhile, our RNA-Seq results found that significantly changed genes with transcriptome were related to energy metabolism. Overall, we found that the grape pomace diet could improve the meat quality and nutrient digestibility of finishing pigs.

Intestinal barrier injury, disturbed gut microbiota, inflammation, and oxidative damage have been widely reported to result in irreversible growth performance (Feng et al., 2021; Zou et al., 2021). The intestinal health, which was perilously juxtaposed between the external environment and the host immune system, which could be influenced by dietary components (Zou et al., 2021). Our results of serum showed that the total antioxidant capacity ability and scavenge superoxide free radicals’ ability of the treatment group were increased, which was beneficial to pigs’ gut health. Studies showed that a diet with phenolic compounds could attenuate oxidative stress in piglets (Hu et al., 2022). Surprisingly, we found that the grape pomace diet could decrease the oxidant stress of finishing pigs. IgA, IgM, IgG, and IFN-γ of the treatment group were significantly increased in our study, and IL-1β of the treatment group was decreased significantly. Immunoglobulin (Ig), including IgA, IgG, and IgM, played an important role in humoral immune performance (Khan et al., 2021). Cytokines were small peptides that were of great importance in the mediation of immune and inflammatory responses (Cook et al., 2018). Over expression of pro-inflammatory (IL-1β, IL-6, and IL-10) factors may cause disease. IFN-γ had antiviral and immune regulation abilities (Cook et al., 2018). Toll-like receptors (TLRs) also worked effectively in the innate immune system (Xie et al., 2019). It was found that the TLRs’ mRNA expression of the ileum and spleen was increased significantly (TLR-1, TLR-5, etc.). Our results showed that the grape pomace diet improved finishing pigs’ immune performance, which may be related to phenolic compounds. The intestinal epithelial barrier, which mainly involved a single layer of epithelial cells that are interconnected by tight junctions, could control the absorption of nutrients and prevent pathogens and toxins from entering the systemic circulation (Peterson and Artis, 2014). In the treatment group, we found a significant increase in the mRNA expression of ZO-1, occludin-1, and claudin-1, which were regarded as a marker of the selectively permeable barrier (Hu et al., 2022). Serum DAO was usually considered as a waft of intestinal damage (Jing et al., 2014). In our results, the treatment group’s DAO content was significantly lower than the control group, which was consistent with our mRNA results. In a word, these results showed that the treatment group attenuated oxidative stress and inflammation and improved the intestinal barrier function.

The intestinal microbiota, which depends mostly on non-digestible fibers and polysaccharides acting as energy sources, played a vital role in many aspects of host physiology (Xu et al., 2013). Metabolic substances deriving from gut microbiota, such as short-chain fatty acids and bacteriocin, could promote gut development and prevent diarrhea (Li et al., 2018). In addition, some studies found that gut microbiota disturbance could induce barrier damage and intestinal inflammation (Li et al., 2018). It had been reported that *Treponema* and *Streptococcus* would make negative differences in pigs, and they could drive swine diarrhea, intestinal inflammation, and other intestinal diseases (Goyette-Desjardins et al., 2014; Burrough, 2017). In our study, a diet with grape pomace significantly decreased the relative abundance of *Treponema* and *Streptococcus*. We found that *Treponema* and *Streptococcus* had a negative linear correlation with immunoglobulin, inflammatory cytokines, and antioxidant indices. It means that the decrease in the relative abundance of *Treponema* and *Streptococcus* may be related to improving the levels of immunoglobulin and antioxidant capacity immune, which could improve immune performance. Our study proved that *Streptococcus* and *Treponema* group had a negative linear correlation with IgA, TNF-γ, and other serum immune performance. It may represent that a diet with grape pomace could decrease intestinal inflammation by decreasing the relative abundance of *Treponema* and *Streptococcus*. Pig health affects the production and quality of meat, and grape pomace has phenolic compounds which could improve meat quality. Grape pomace could improve immunity, these may be the main reason that grape pomace could improve meat quality. Based on our results, the grape pomace diet decreased the relative abundance of *Treponema* and *Streptococcus*, which may have potential benefits for meat quality, antioxidant capacity, and immune performance in finishing pigs. In short, our study found that grape pomace could change the gut microbiota of finishing pigs, and it may improve immune performance by decreasing the relative abundance of *Treponema* and *Streptococcus.*

## Conclusion

In summary, the wheat bran of diet replaced with grape pomace could improve the immune performance in finishing pigs. Through the analysis of microbiota, we found that *Treponema* and *Streptococcus* may affect the immune performance and antioxidant capacity of finishing pigs. Our results could provide a novel strategy for reducing feeding costs and improving the immune performance in finishing pigs. However, the functions of grape pomace that improve the immune performance by decreasing the relative abundance of *Treponema* and *Streptococcus* await further investigation.

## Data availability statement

The data presented in the study are deposited in the NCBI’s archival repository, accession number PRJNA923828.

## Ethics statement

The animal study was reviewed and approved by Animal Care and Use Committee of Northwest A&F University (Yangling. China). Written informed consent was obtained from the owners for the participation of their animals in this study.

## Author contributions

TYu conceived and designed the experiment. XT, DL, XZh, and ZX performed experiments, analyzed data, and wrote the manuscript. JS and TYua assisted part of the experiment and the modification of the article. XZu and YW provided the grape pomace used in this article. GY contributed to the revisions. All authors contributed to the article and approved the submitted version.

## Funding

This study was supported by the National Key R&D Program of China (2021YFD1301200), the Key Research and Development Program of Shaanxi Province (2023-YBNY-106) and the Shaanxi Livestock and Poultry Breeding Double-chain Fusion Key Project (2022GD-TSLD-46).

## Conflict of interest

YW was employed by Qinghai Yufu Animal Husbandry Development Co., Ltd. XZu was employed by Ningxia Lilan Winery Co., Ltd.

The remaining authors declare that the research was conducted in the absence of any commercial or financial relationships that could be construed as a potential conflict of interest.

## Publisher’s note

All claims expressed in this article are solely those of the authors and do not necessarily represent those of their affiliated organizations, or those of the publisher, the editors and the reviewers. Any product that may be evaluated in this article, or claim that may be made by its manufacturer, is not guaranteed or endorsed by the publisher.

## Supplementary material

The Supplementary material for this article can be found online at: https://www.frontiersin.org/articles/10.3389/fmicb.2023.1116022/full#supplementary-material
