# Supplementary figures and images for: Dietary grape pomace extract supplementation improved meat quality, antioxidant capacity, and immune performance in finishing pigs
Source: Front Microbiol. 2023 Mar 2;14:1116022. doi: 10.3389/fmicb.2023.1116022 (PMC10017996; doi:10.3389/fmicb.2023.1116022)

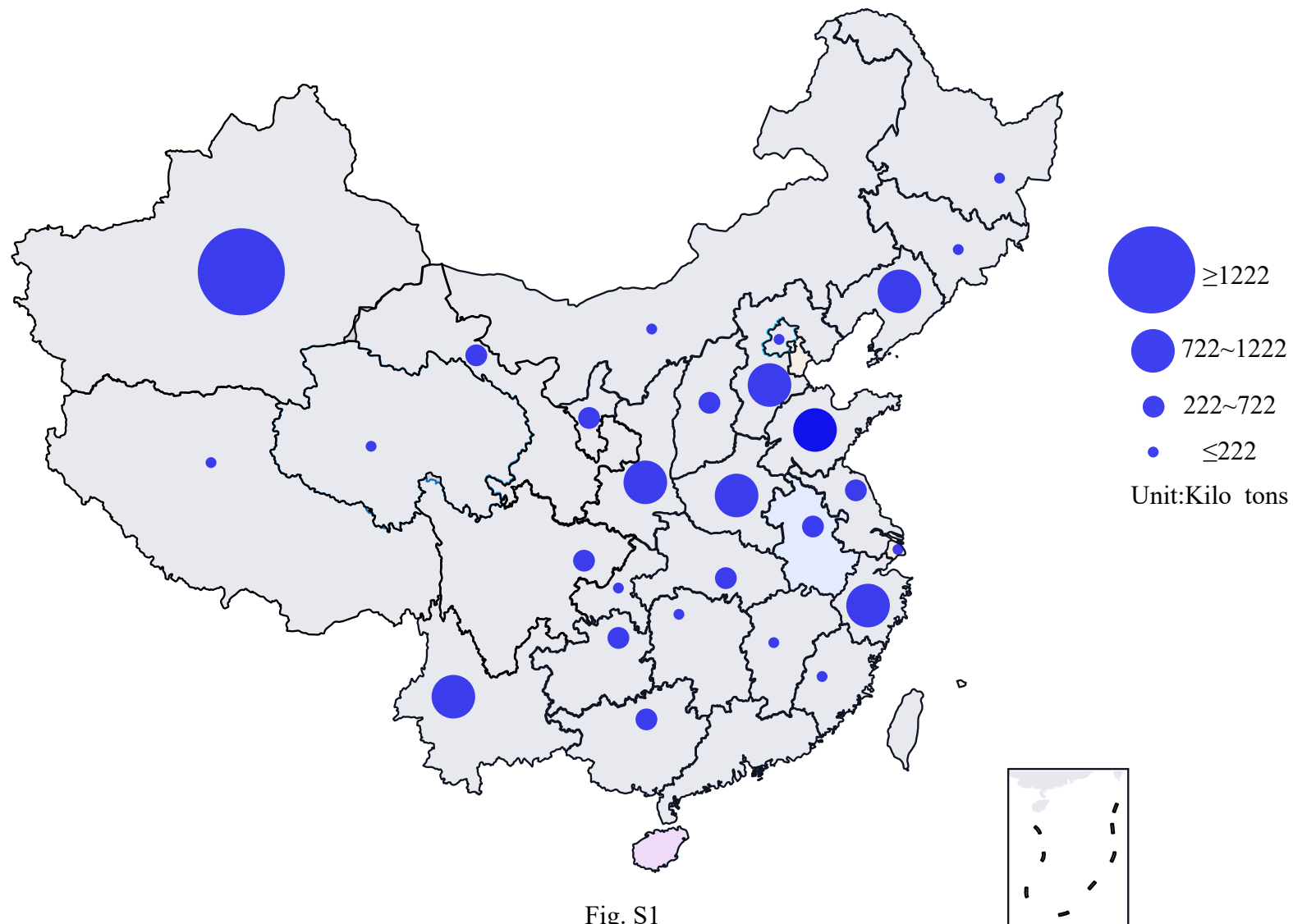

Supplement: Supplementary file 1 [file Image_1.PDF]

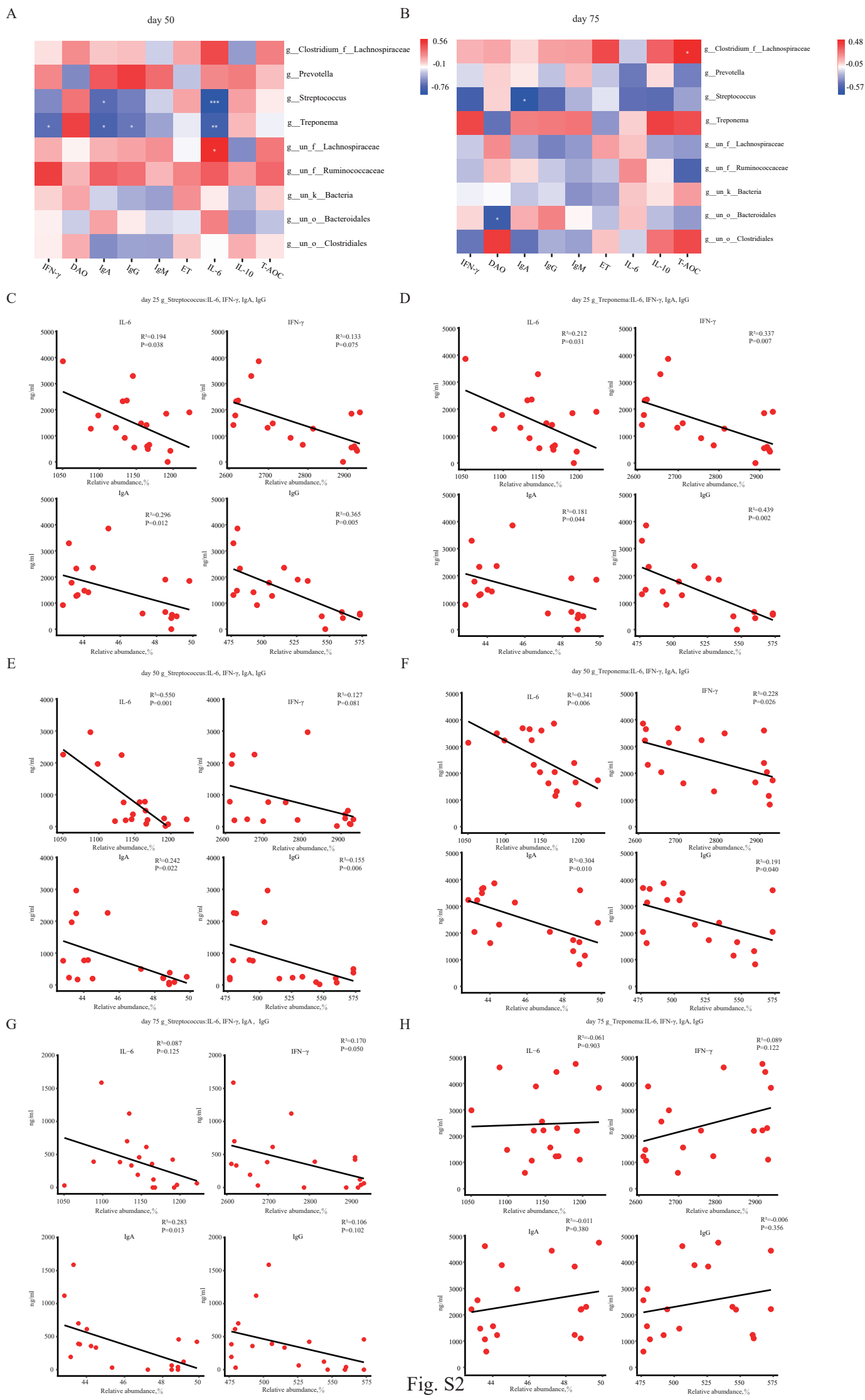

Fig. S2

Supplement: Supplementary file 2 [file Image_2.PDF]
