# Supplementary material for: Dietary grape pomace extract supplementation improved meat quality, antioxidant capacity, and immune performance in finishing pigs
Source: Front Microbiol. 2023 Mar 2;14:1116022. doi: 10.3389/fmicb.2023.1116022 (PMC10017996; doi:10.3389/fmicb.2023.1116022)
Supplement: Supplementary file 3 [file Table_1.DOCX]

| Items | Control | Treatment |
| --- | --- | --- |
| Initial weight (kg) | 54.72±3.73 | 55.56±4.30 |
| Final weight (kg) | 115.28±6.48 | 115.11±10.19 |
| ADG (kg) | 0.71±0.06 | 0.72±0.10 |
| ADFI (kg) | 2.48±0.17 | 2.38±0.27 |
| F:G (kg/kg) | 3.52±0.39 | 3.34±0.41 |
| Hot carcass weight (kg) | 87.74±5.09 | 88.60±7.56 |
| Dressing percentage (%) | 76.13±2.00 | 77.05±3.31 |
| Loin muscle area (cm^2^) | 30.98±6.21 | 29.06±4.31 |

Table 1 Effect of grape residue meal on carcass traits of longissimus thoracis in pigs.

Note: a,b Means in the same row with no superscript letters after them or with a common superscript letter following them are not significantly different (*p>0.05*). Values are expressed as the means ± SD (n = 6); ADG: daily weight gain, ADFI: average daily feed intake, F:G: feed conversion ratio.
