# Supplementary material for: Dietary grape pomace extract supplementation improved meat quality, antioxidant capacity, and immune performance in finishing pigs
Source: Front Microbiol. 2023 Mar 2;14:1116022. doi: 10.3389/fmicb.2023.1116022 (PMC10017996; doi:10.3389/fmicb.2023.1116022)
Supplement: Supplementary file 4 [file Table_2.DOCX]

Table 2 Effect of grape residue meal on quality traits of longissimus thoracis in pigs.

| Items | Control | Treatment |
| --- | --- | --- |
| pH_45min_ | 6.63±0.13 | 6.78±0.10 |
| L* _45min_ | 44.52±1.19 | 45.06±1.34 |
| a*_45min_ | 8.15±0.69 | 8.21±0.48 |
| b*_45min_ | 15.42±0.33 | 15.78±1.02 |
| pH_24h_ | 5.58±0.09 | 5.68±0.03 |
| L* _24h_ | 53.51±0.94 | 54.50±0.91 |
| a*_24h_ | 11.37±2.27 | 12.72±1.11 |
| b* _24h_ | 19.60±0.98 | 19.61±1.40 |
| Water loss _24h_ (%) | 32.90±2.81^a^ | 24.82±1.33^b^ |
| Drip loss _24h_ (%) | 2.54±0.19^a^ | 1.49±0.10^b^ |
| Water loss _48h_ (%) | 38.03±1.73^a^ | 33.31±2.02^b^ |
| Drip loss _48h_ (%) | 7.16±0.49^a^ | 4.32±0.23^b^ |
| Share Force (N) | 31.81±7.66 | 39.63±6.37 |
| Cooking loss (%) | 35.25±0.83^a^ | 32.05±0.79^b^ |

Note: a,b Means in the same row with no superscript letters after them or with a common superscript letter following them are not significantly different (*p*>*0.05*). Values are expressed as the means ± SD (n = 6).
